# Dysregulation of microRNAs and renin-angiotensin system in high salt diet-induced cardiac dysfunction in uninephrectomized rats

**DOI:** 10.1371/journal.pone.0180490

**Published:** 2017-07-20

**Authors:** Venkateswara Rao Amara, Sunil Kumar Surapaneni, Kulbhushan Tikoo

**Affiliations:** Laboratory of Epigenetics and Diseases, Department of Pharmacology and Toxicology, National Institute of Pharmaceutical Education and Research (NIPER), S.A.S. Nagar, Punjab, India; Max Delbruck Centrum fur Molekulare Medizin Berlin Buch, GERMANY

## Abstract

Uninephrectomy is not associated with major adverse events in cardiovascular and renal functions of live kidney donors. The effect of high salt diet on the quality of life of live kidney donors is largely unknown. Hence in this study, we aimed to determine the effect of high salt diet on the alterations of renin-angiotensin system and microRNAs leading to CV and renal dysfunction in uninephrectomized rats. In order to mimic clinical scenario, uninephrectomized male Sprague Dawley rats were fed initially with normal pellet diet for 12 weeks and then for 20 weeks with high salt (10% w/w NaCl) diet. At the end of the study, biochemical, functional, histological and molecular parameters were measured. High salt diet feeding resulted in renal dysfunction & fibrosis, decreased baroreflex sensitivity, increased *in vivo* cardiovascular reactivity to angiotensin II owing to upregulation of angiotensin II type 1 receptors and L-type calcium channels leading to cardiovascular dysfunction in uninephrectomized rats (UNX+HSD) worse than that of normal (binephric) rats fed with high salt diet (HSD). Protein expression of functional and hypertrophic protein markers revealed decreased SERCA, p-AMPK and increased p-AKT. Interestingly, levels of miR-25, miR-451 and miR-155 increased and miR-99 decreased in heart of uninephrectomized rats fed with high salt. However, circulating miR-25 and miR-451 levels decreased and miR-99b increased in these animals. Our study points out that since tissue and circulating levels of miRNAs are not similar, caution must be exercised during the usage of miRs as diagnostic or prognostic biomarkers. To our knowledge, we are the first to show that epigenetic alterations result in cardiac dysfunction in uninephrectomized rats fed with high salt diet.

## Introduction

Globally, cardiovascular diseases (CVDs)—ischemic heart disease and cerebrovascular disease are the number-one and two death-causing diseases for the past decade [1, 2]. With the exception of very insignificant genetic predisposition, the risk factors of CVDs are lifestyle-acquired including increased intake of salt, fructose, fat-rich foods, alcohol, smoking, physical inactivity, stress, etc. [3, 4].

Patients with both the kidneys in critical condition as in chronic end-stage renal disease, uncontrolled renal calculi etc., require a healthy kidney from living donors, rather than cadavers [5, 6]. Majority of the reports did not find any major renal or cardiovascular complications in donor nephrectomized people even after many years of surgery [7, 8]. However, there are no reports showing the effect of diet-induced renal or cardiovascular complications in these donor nephrectomized patients.

High salt consumption causes pressure-dependent and independent cardiovascular and renal complications [9]. In Indian diet, high salt is consumed in the form of salt used in cooking, pickles, fast foods, junk foods, processed and ready-made foods, salt added at the table directly [10]. Since Salt loading is known to affect renin-angiotensin system (RAS) [11], we hypothesized that the concomitant condition of uninephrectomy (reduced nephron number) and high salt intake may aggravate CV and renal complications. In addition, to our knowledge, so far, no data is available regarding the activity of local RAS (kidney, heart and blood vessels) under high salt intake in uninephrectomy condition. Hence to address this, in the present study, we used *in vivo* Ang II-induced cardiovascular reactivity (rather than conventional *ex vivo* organ bath for blood vessel and Langendorff system for heart).

Several reports suggest the role of miRNAs in diseases like cancer, metabolic, inflammatory, neurological and cardiovascular diseases [12]. Circulating miR-208a and miR-126 were found to be significantly and consistently altered in the plasma under different cardiac pathological conditions, like acute myocardial infarction, heart failure and coronary artery disease [13]. Roles of miR-25 [14], miR-155 [15] & miR-451 [16] in various murine CVDs have been reported but their role in high salt diet-induced CV complications in uninephrectomized rats is not yet reported. High salt diet is known to induce cardiac hypertrophy and fibrosis [17, 18] but its effect on CV and renal functions in uninephrectomy is not known. In order to get insight into the quality of life of live kidney donors consuming high salt, the present study was designed to study the involvement of RAS and miRs in mediating CV and renal dysfunction.

## Materials and methods

### Animals and experimental design

Adult male Sprague Dawley rats of 200-250g body weight were procured from the Central Animal Facility of the institute and housed 3 rats/cage under standard environmental conditions (temperature: 20 ± 1°C, humidity: 50 ± 10%; and 12 h light/dark cycle) with access to food and water *ad libitum*. All protocols were approved by the Institutional Animal Ethics Committee and experiments performed in accordance with the guidelines of the Committee for the Purpose of Control and Supervision of Experiments on Animals (CPCSEA) (IAEC 12/11), India and complied with the NIH guidelines (Guide for the care and use of laboratory animals). All the studies involving animals were reported in accordance with ARRIVE guidelines [19]. All the groups were planned in such a way that each group after diet switching from normal pellet diet to high salt diet would contain 5–6 animals and no mortality was observed during surgery.

Animals were fed normal pellet for 12 weeks after UNX surgery to mimic the actual clinical setting in which donor nephrectomised people will be on strict diet control at least for a certain period after surgery upon physician/surgeon’s advice. Sham surgery was performed in the Control animals in the same manner of uninephrectomy surgery except that ligations and excision of kidneys were not performed. Then normal and uninephrectomized animals were randomized into 4 groups—normal control (Ctrl) and uninephrectomized rats (UNX) fed with normal pellet diet, normal (HSD) and uninephrectomized rats fed with high salt diet (10% w/w NaCl) (UNX+HSD) [20, 21] for 20 weeks till the end of the experiment. At the end of the study, immediately after performing haemodynamic experiments using urethane anesthesia, animals were euthanized by exsanguination and organs were collected for further experiments.

### Uninephrectomy surgery

Animals were injected with saline (20 ml/kg, s.c.) to prevent the fluid loss due to evaporation from cut ends or the peritoneal fluid loss during laparotomy [22] and then anaesthetized using ketamine + xylazine (70 mg/kg + 7 mg/kg, i.p). After the loss of pedal pain, corneal reflexes, a half-inch incision was given on the left flank portion of abdomen and kidney was pulled out of the abdomen by holding the perirenal fat at the lower pole with blunt forceps, separating kidney from the surrounding fat and supra renal gland, ligation of renal artery & vein and ureter ~0.5cm below the level of hilum with a non-absorbable surgical suturing thread and kidney was snap resected. Then skeletal muscle and skin layers were sutured separately with absorbable and non-absorbable sutures respectively and the animal was allowed to recover by placing in a solitary cage under the heat of 60W lamp.

After nephrectomy, topical (Betadine^™^) antiseptic and parenteral (Augmentin^™^, 324 mg/kg, i.p.) antibiotics were given to prevent postsurgical infection and analgesic to alleviate pain. Antiseptic was applied daily for 7–10 days for the effective healing of the surgical incision.

### Biochemical parameters

Animals were fasted overnight; blood was collected from tail vein and centrifuged at 4°C, 2500×g for 10 mins for separation of plasma. Different biochemical parameters were measured as per the manufacturer’s instructions. Glucose (GOD-POD), lipid profile—triacylglycerols (LPL-GK-GPO-POD), cholesterol (CHE-CHO-POD), kidney function tests (KFT)—albumin (BCG), blood urea nitrogen (BUN) (Urease), creatinine (Jaffe’s initial rate method) (Accurex Biomedical Pvt. Ltd., Mumbai, India; Crest Biosystems, Goa, India), creatinine in urine (Jaffe’s end-point method), sodium (Uranyl acetate/ferrocyanide method), potassium (Turbidometry) (Crest Biosystems, Goa, India) and angiotensin II (Competitive-ELISA) (Elabscience Biotechnology Co. Ltd, WuHan, China).

### Haemodynamic parameters—Basal and stimulated

Rat was injected with urethane (1.2 g/kg, i.p) and anesthesia was confirmed with the loss of pedal, tail pain and corneal reflexes. Animal was restrained in its supine position by fastening the limbs on a dissection board maintained at a constant temperature of 37°C. Neck region of the animal was clipped off hair, disinfected with 70% alcohol. Location of left jugular vein was felt with the pulse felt at the level of clavicle (collar bone), exposed and cannulated for administration of drugs for *in vivo* vascular reactivity measurement. Then, an incision of ~1 cm in the neck just to the right of sagittal axis, and right carotid artery was exposed by blunt dissection of the para tracheal muscles and a carotid arterial intubation was done with a heparinised (40 IU/mL) saline—filled cannula fitted with a 3-way stopcock to a pressure transducer (MLT844 Physiological Pressure Transducer, ADInstruments, Australia), previously calibrated, and connected to the data acquisition system (PowerLab, ADInstruments, Australia), which is connected to a computer where Invasive Blood Pressure (IBP) is displayed. After 20 mins of stabilisation period, acute changes in mean arterial pressure (MAP) to graded doses of angiotensin II (Sigma-Aldrich) were taken in order—20, 40, 80, 160, 320 ng/kg with relaxation period between the successive doses. Relaxation period was taken as the time for the MAP to return to basal level.

For measurement of left ventricular systolic pressure (LVSP), heparinised (40 IU/ mL) saline—filled cannula in the right carotid artery was advanced carefully into the left ventricle till the increased magnitude of invasive blood pressure (with max. SBP and DBP ~0 mm Hg) signal observed on the PC attached to the Data acquisition system (PowerLab, ADInstruments, Bella Vista, Australia). Basal LVSP was taken after a stabilisation period of 10 mins and *in vivo* cardiac reactivity to Ang II (20, 40, 80, 160, 320 ng/kg) was measured giving relaxation between the successive doses. Relaxation period was taken as the time for the LVSP to return to basal level. Haemodynamic data was analysed with LabChart 7 (ADInstruments, Australia).

Acute response of the heart and blood vessel to 160 μg/kg diltiazem (DTZ) (Marion Labs) and mixture of diltiazem and 160 ng/kg angiotensin II was measured by injecting bolus doses and quantified in the manner same as that followed for Ang II.

### Baroreflex sensitivity (BRS)

Baroreflex sensitivity (BRS) was measured as described by Khaliq et al. by administering increasing doses of vasoconstrictor phenylephrine (10, 20, 30 μg/kg) (Sigma-Aldrich) as bolus ramp infusions [23]. The duration of drug infusion and relaxation period (5 mins) between consecutive doses were kept constant for all the animals. The resultant change in heart rate (ΔHR) i.e., bradycardia at corresponding increment in mean arterial pressure (ΔMAP) was measured and ΔHR/ΔMAP (beats/min/mmHg), the index of BRS was plotted against the dose of phenylephrine.

### Histology and immunohistochemistry

Thoracic aorta, kidney and heart were fixed in 10% v/v formal saline, embedded in paraffin, 5 μm transverse sections were prepared and mounted on slides previously coated with Mayer’s albumin. Sections were stained with hematoxylin & eosin for structural changes and picrosirius red, which selectively stains collagen red, for fibrosis. For immunohistochemistry, paraffin sections were processed, antigen was retrieved by incubating the slides in EDTA buffer, pH 8 at 95°C for 15 mins, peroxidase blocked with 3% v/v H_2_O_2_, blocked with 3% w/v bovine serum albumin to prevent non-specific binding and incubated with primary antibody (sc-1173, 1:50 v/v dilution) and anti-rabbit IgG-HRP labelled secondary antibody and developed using 3,3’-diaminobenzidine (Novolink^™^ DAB, Cat# RE7230-K, Leica Biosystems Newcastle Ltd, UK) which renders the protein of interest positive areas brown. Coverslip was mounted with the help of DPX and observed at 400X, 1000X magnification using OLYMPUS BX51 microscope and the images were captured with OLYMPUS DP 72 camera attached to the microscope. Histological images were blinded and quantified using ImageJ software (NIH, USA).

### Western blotting of heart and kidney

Protein isolation and western blotting was performed as previously described [24]. Briefly, left ventricle of hearts and kidneys were thawed, minced and homogenized in lysis buffer containing surfactants, protease and phosphatase inhibitors. Protein samples were resolved using 8% and 10% w/v sodium dodecyl sulfate-polyacrylamide gels depending on the molecular weight of desired proteins. These were then electrotransferred to nitrocellulose membranes and were incubated with below mentioned antibodies—ACE2 (rabbit pAb, Cat# sc-20998), p-AMPK (rabbit pAb, sc-33524), AMPK (rabbit pAb, sc-25792), p-AKT (Rabbit pAb, SAB4300042), AKT (rabbit pAb, SAB4500802), actin (goat pAb, sc-1616, SantaCruz Biotechnology, Inc., CA, USA), SERCA = ATP2A2 (rabbit pAb, Cat# HPA062605-100UL, Sigma-Aldrich, MO, USA). The antigen-primary antibody complexes were incubated with horseradish peroxidase (HRP)-conjugated secondary antibodies (SantaCruz Biotechnology, Inc., CA, USA) and were visualized using enhanced chemiluminescence substrate (Invitrogen, CA, USA) and ECL hyperfilm (GE Healthcare Pvt. Ltd., UK). Blots were scanned and analyzed using ImageJ software (NIH, USA).

### RT-qPCR of mRNA in heart

Total RNA was isolated from heart by homogenising and extracting in TRIzol Reagent (Invitrogen, ThermoFisher Scientific, CA, USA), chloroform/isopropanol, precipitated and dispersed in RNAse-free water. The quality and integrity of RNA was assured by measuring 260/280 ratio (NanoDrop ND-100, Thermo Scientific, USA) and running agarose gel electrophoresis respectively. Reverse transcription of mRNA was performed using commercial cDNA synthesis kit (Verso cDNA synthesis kit, Thermo Scientific, Cat# AB-1453/A). Quantitative real-time PCR was performed in LightCycler 2.0 (Roche, Switzerland) using SYBR green master mix (SYBR Premix Ex Taq II, Cat#RR820A, TaKaRa Bio Inc., Otsu, Japan,), specific forward and reverse primers designed by NCBI Primer-BLAST (Agtr1a: Forward- 5’-GGATTCGTGGCTTGAGTCCT-3’, Reverse- 5’-TCACTTTCTGGGAGGGTTGT-3’; Agtr2: Forward- 5’-GAACAGAATTACCCGTGACCA-3’, Reverse- 5’-ATGAATGCCAACACAACAGC-3’), synthesized commercially (Eurofins Scientific, Bengaluru, India) and the specificity of reaction was assessed by analysing melting curve of PCR product. The relative gene expression was quantified by 2^-ΔΔCt^ method and normalised with18S rRNA.

### RT-qPCR of miRNA in heart and plasma

Left ventricle portion of heart was homogenized in a proprietary buffer and miRNA was isolated using commercially available kits (PureLink^™^ miRNA Isolation kit, Cat# K1570-01, Invitrogen, CA, USA for heart and miRCURY RNA Isolation Kit-Biofluids, Cat#300112, Exiqon, MA, USA for plasma), cDNA was synthesized using miRCURY LNA^™^ Universal RT microRNA PCR, Cat#203301, Exiqon, MA, USA in 2720 Thermal Cycler (Applied Biosystems, CA, USA), purified using PureLink^™^ Quick PCR Purification Kit, (Cat# K310001,Invitrogen, Poststraβe, Germany) and PCR was run in LightCycler 2.0 (Roche, Basel, Switzerland) using readymade specific forward and reverse primer mix (TaqMan^®^ MicroRNA Assays, Applied Biosystems, CA, USA; microRNA LNA^™^ PCR primer sets, Exiqon, MA, USA) and the amplification curves were analyzed using 2^-ΔΔCt^ method. Micro RNAs quantified were hsa-miR-25-3p (Cat# 204361), mmu-miR-155-5p (Cat# 205930), hsa-miR-99b-3p (Cat# 204064) and mmu-miR-451(Cat# 204734) and normalized with RNU5G (a small nuclear RNA) in heart and hsa-miR-30e-5p (Cat# 204714) in plasma.

### Statistical analysis

All the data were expressed as mean ± SEM. For determining statistical significance, means of two groups, were compared using t-test and multiple groups using one way ANOVA followed by Bonferroni post hoc test. For analyzing baroreflex sensitivity and *in vivo* cardiovascular reactivity to angiotensin II, phenylephrine data, two-way ANOVA followed by Bonferroni post hoc test were used. Values were considered statistically significant if P<0.05. Statistical software used was GraphPad Prism 5.01.

## Results

### High salt diet deranges kidney function and instigates renal and cardiac hypertrophy

There was no significant difference between normal control (Ctrl) and uninephrectomized (UNX) rats in terms of % BW change, plasma albumin, creatinine as long as the animals were on normal diet (Table 1). After 20 weeks of high salt diet feeding, polyphagia, polydipsia, polyuria, wasting, lipolysis, increased systemic angiotensin II, renal dysfunction & cardiac hypertrophy were observed in rats fed with high salt diet (HSD) and uninephrectomized rats fed with high salt diet (UNX+HSD) compared to Ctrl and UNX groups (Table 2).

**Table 1:** General parameters, plasma kidney function tests, glycemic and lipid profile post-uninephrectomy during normal pellet diet feeding.

Time point	Week 4		Week 8		Week 12	
Parameter	Ctrl	UNX	Ctrl	UNX	Ctrl	UNX
% wt. change	43.85 ± 3.02	51.86 ± 2.70	64.27 ± 3.89	73.83 ± 4.48	74.8 ± 4.60	78.43 ± 7.03
Albumin (μmol/L)	435 ± 20.3	421.95 ± 7.25	507.5 ± 15.95	484.3 ± 13.05	532.15 ± 4.35	533.6 ± 4.35
BUN (mmol/L)	5.83 ± 1.36	6.18 ± 0.84	5.13 ± 0.74	5.40 ± 0.39	6.20 ± 0.42	7.34 ± 0.45
Creatinine (μmol/L)	69.83 ± 1.77	69.84 ± 6.19	97.24 ± 16.80	125.53 ± 14.14	87.516 ± 7.80	99 ± 5.304
BUN/Creatinine	17.76 ± 1.57	20.62 ± 2.48	15.81 ± 0.57	13.78 ± 0.81	18.29 ± 1.58	19.22 ± 1.75

Values were mean ± S.E.M of 10–12 animals in Ctrl and 12 animals in UNX. Ctrl, normal control rats; UNX, uninephrectomized group.

**Table 2:** General parameters, kidney function tests, glycemia, lipids, systemic RAS, morphometry at the end of the study after 20 weeks of high salt diet feeding.

Parameter	Ctrl	HSD	UNX	UNX+HSD
Feed intake (g/24 h)	22 ± 1.155	55 ± 14*	24 ± 0.5774	36.67 ± 4.667*
% ΔBW	24.15 ± 2.638	2.591 ± 4.224**	11.6 ± 3.76	-8.609 ± 2.753***^U^
Water intake (mL/24 h)	37.67 ± 3.18	111.3 ± 18.17**	24 ± 0.5774	191.3 ± 6.25***^SS^^UUU^
Urine output (mL/24 h)	10 ± 1	38.83 ± 5.51*	15.5 ± 3.5	79 ± 19**^S^^U^
Plasma albumin (μmol/L)	527.8 ± 7.25	526.35 ± 7.25	479.95 ± 20.3	493 ± 8.7
Plasma BUN (mmol/L)	4.82 ± 0.30	8.06 ± 0.11***	8.32 ± 0.42***	10.45 ± 0.39***^SSS^^UUU^
Plasma Creatinine (μmol/L)	132.6 ± 11.5	213.04 ± 20.33*	122.88 ± 18.56	217.46 ± 18.56*^U^
Urine albumin (μmol/L)	906.25 ± 189.95	977.3 ± 108.75	1041.1 ± 336.4	887.4 ± 308.85
Urine UN (mmol/L)	1157.03 ± 61.18	299.45 ± 39.95***	1156.32 ± 81.57	126.38 ± 11.74***^UUU^
Urine Creatinine (μmol/L)	16283.28 ± 926.43	3610.26 ± 1749.44***	12915.24 ± 2096.85	987.43 ± 204.20***^UU^
Urine sodium (mmol/L)	718.5 ± 78.51	1590 ± 71.41*	1134 ± 139.20	2088 ± 188.10***^S^^UU^
Urine potassium (mmol/L)	379.6 ± 15.39	184.3 ± 6.59**	371.5 ± 55.22	70.67 ± 4.43***^UUU^
Plasma Glucose (mmol/L)	6.41 ± 0.22	6.23 ± 0.16	6.56 ± 0.15	5.97 ± 0.29
Plasma triacylglycerols (mmol/L)	0.71 ± 0.03	0.37 ± 0.04**	0.67 ± 0.14	0.31 ± 0.02***^U^
AUC of IPGTT (mmol/L*min)	1202.6 ± 82.88	1055.38 ± 39.68	1078.73 ± 104.83	1037.62 ± 293.39
Plasma Angiotensin II (pmol/L)	409.70 ± 55.35	174.46 ± 21.91***	262.51 ± 43.58	315.41 ± 18.76***^UU^
Kidney wt. (g)	1.42 ± 0.04	1.45 ± 0.11	1.95 ± 0.07**	2.17 ± 0.13**^SS^
Kidney wt. index (mg/g)	2.79 ± 0.13	3.68 ± 0.23*	4.64 ± 0.05***	5.81 ± 0.11***^SSS^^UU^
Heart wt. (g)	1.36 ± 0.04	1.25 ± 0.07	1.24 ± 0.13	1.20 ± 0.06
Heart wt. index (mg/g)	2.64 ± 0.03	3.17 ± 0.15**	3.01 ± 0.10	3.28 ± 0.14*

Values were mean ± S.E.M of 5–6 animals in all the groups. Ctrl, normal control rats; HSD, normal rats fed with high salt diet; UNX, uninephrectomized rats; UNX+HSD, uninephrectomized rats fed with high salt diet.

*P<0.05,

**P<0.01,

***P<0.001 vs. Control;

^S^P<0.05,

^SS^P<0.01,

^SSS^P<0.001 vs HSD;

^U^P<0.05,

^UU^P<0.01,

^UUU^P<0.001 vs. UNX.

### High salt diet worsens basal haemodynamics in uninephrectomized rats

**Fig 1:**
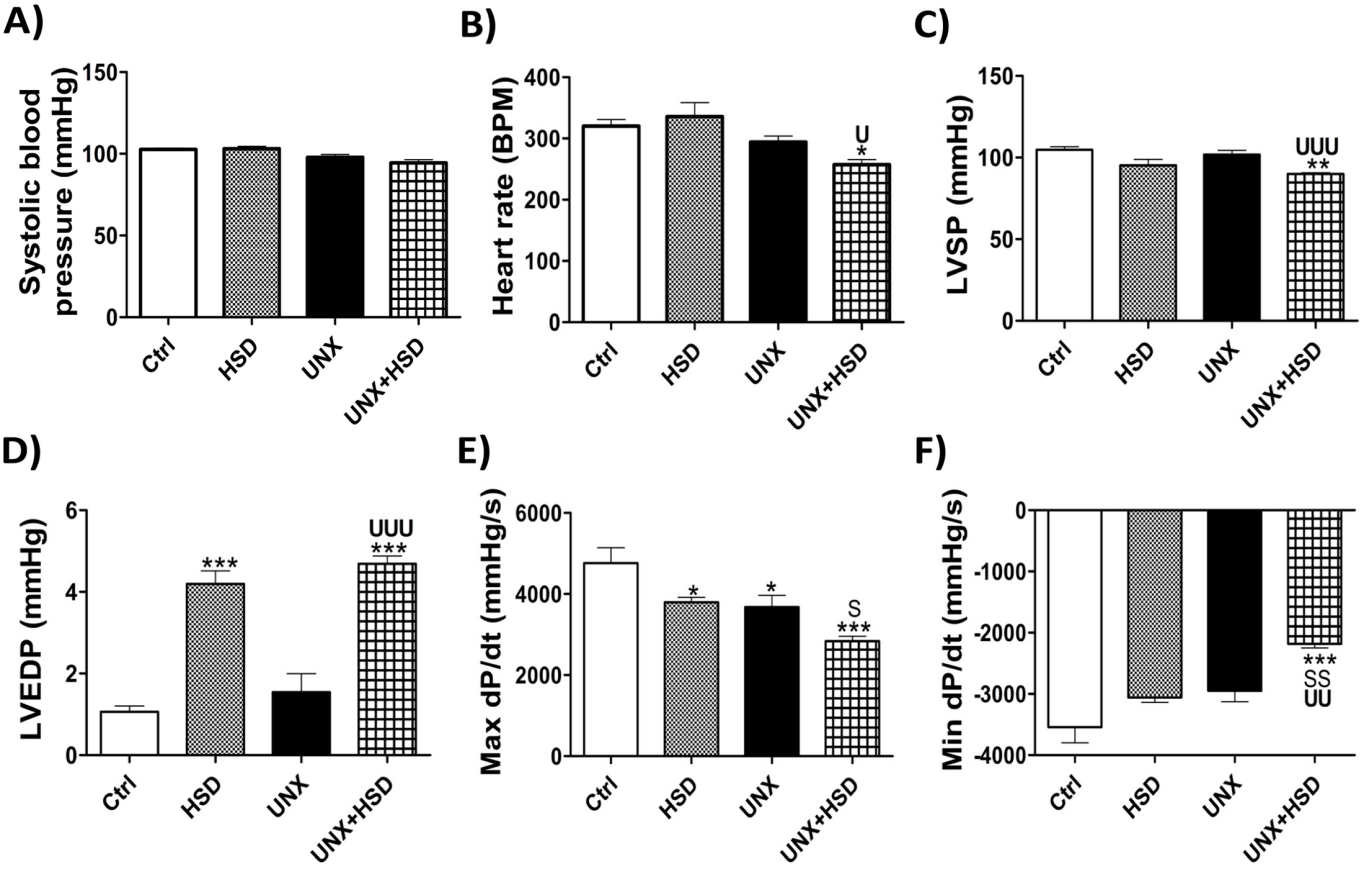
Basal haemodynamic parameters. A) SBP, B) HR, C) LVSP, D) LVEDP, E) +dP/dt, F)–dP/dt. SBP, systolic blood pressure; HR, heart rate; LVSP, left ventricular systolic pressure; LVEDP, left ventricular end- diastolic pressure; +dP/dt, maximum rate of LVP rise during isovolumetric contraction; -dP/dt, maximum rate of LVP decay during isovolumetric relaxation. Ctrl, normal control rats; HSD, normal rats fed with high salt diet; UNX, uninephrectomized rats; UNX+HSD, uninephrectomized rats fed with high salt diet. N = 5–6; *P<0.05, **P<0.01, ***P<0.001 vs. Control; ^S^P<0.05, ^SS^P<0.01 vs. HSD; ^U^P<0.05, ^UU^P<0.01, ^UUU^P<0.001 vs. UNX.

Systolic blood pressure remained unchanged in all the groups despite feeding high salt diet to normal binephric (HSD) and uninephrectomized rats (UNX+HSD) (Fig 1A). Heart rate (HR), left ventricular systolic pressure (LVSP) and maximum rate of LVP decay during isovolumetric relaxation (-dP/dt) decreased significantly in uninephrectomized rats fed with high salt diet (UNX+HSD) compared to Ctrl and UNX (Fig 1B, 1C and 1F). Except +dP/dt, all other parameters of haemodynamics were similar in Ctrl and UNX groups. High salt diet significantly elevated left ventricular end diastolic pressure (LVEDP) and reduced maximum rate of left ventricular pressure (LVP) rise during isovolumetric contraction (+dP/dt) in rats fed with high salt diet (HSD), UNX+HSD groups compared to their respective control animals (Fig 1D and 1E) indicating worsened CV function.

### Parasympathetic component of baroreflex sensitivity (BRS) is altered by high salt feeding

**Fig 2:**
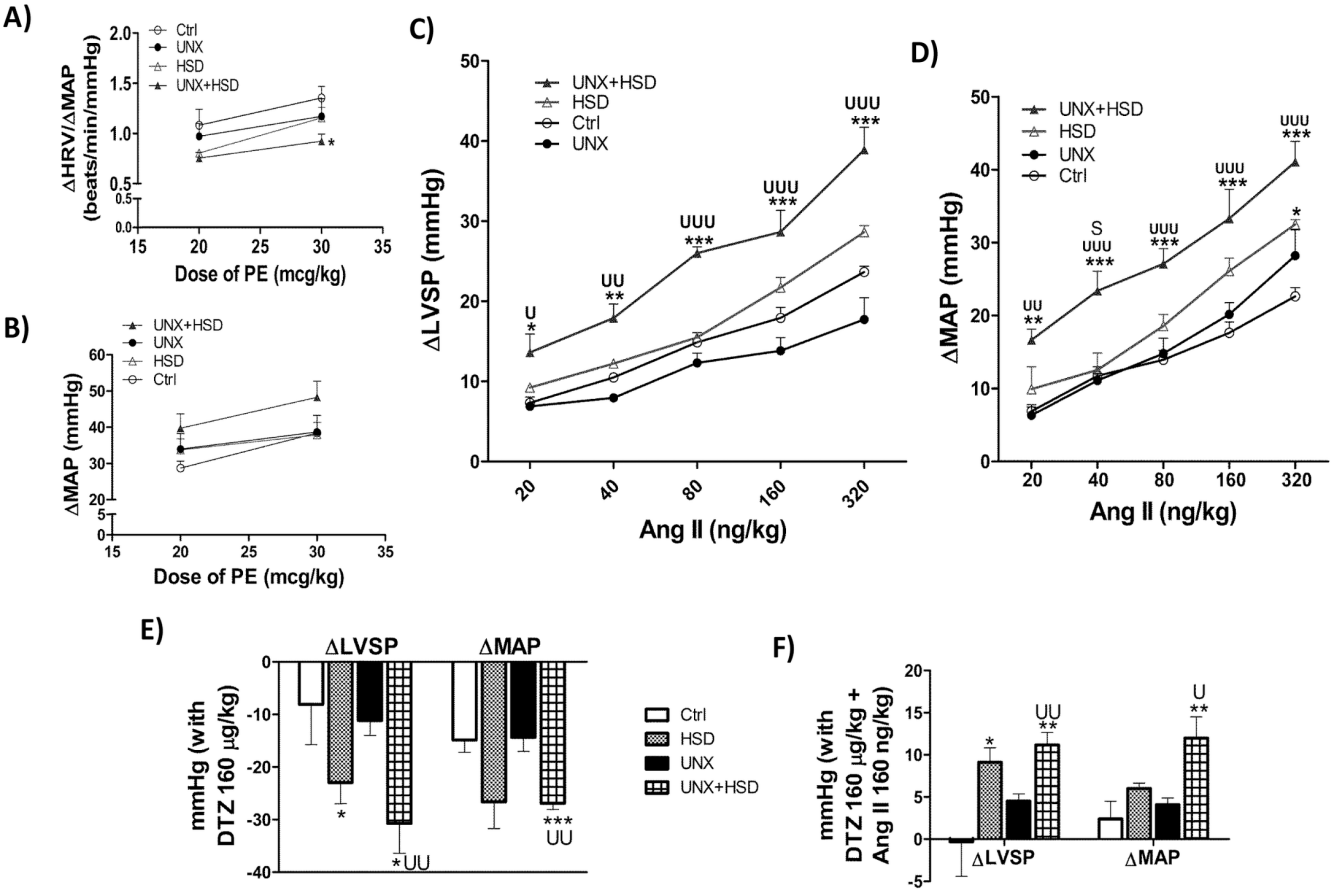
*In vivo* cardiovascular responses to phenylephrine, Ang II and DTZ. A) Parasympathetic component of baroreflex sensitivity measured using phenylephrine, a selective α1-adrenoceptor agonist at doses of 20 and 30 μg/kg. B) In vivo vascular reactivity to phenylephrine. C) In vivo cardiac reactivity to Ang II (20–320 ng/kg), D) In vivo vascular reactivity to Ang II (20–320 ng/kg). E) In vivo cardiac and vascular responses to DTZ (160 μg/kg) respectively. F) In vivo cardiac and vascular response to DTZ (160 μg/kg) + Ang II (160 ng/kg) respectively. Ctrl, normal control rats; HSD, normal rats fed with high salt diet; UNX, uninephrectomized rats; UNX+HSD, uninephrectomized rats fed with high salt diet. ΔLVSP, change in left ventricular systolic pressure, ΔMAP, change in mean arterial pressure; Ang II, Angiotensin II; N = 4; *P<0.05, **P<0.01, ***P<0.001 vs. Control; ^S^P<0.05 vs. HSD; ^U^P<0.05, ^UU^P<0.01, ^UUU^P<0.001 vs. UNX.

BRS is helpful in assessing the development and progression of CVDs [25]. Parasympathetic component of the baroreflex was measured as the ratio of reflex bradycardia response to dose-dependent increase in mean arterial pressure (ΔHR/ΔMAP) by phenylephrine. The slope of BRS plot significantly decreased indicating aberrant parasympathetic activity (vagal stimulation) in the heart of UNX+HSD rats (Fig 2A). Uninephrectomy or high salt diet alone did not bring any excursion in the BRS or ΔMAP in response to phenylephrine. Though the phenylephrine-induced increase in MAP was not statistically significant, it was frank indicating escalated adrenergic nervous system in vasculature (Fig 2B).

### High salt feeding increases in vivo acute cardiovascular reactivity to angiotensin II

In order to check the activity of local RAS in vasculature and heart, we measured the change in mean arterial pressure (ΔMAP) and left ventricular systolic pressure (ΔLVSP) in response to acute bolus doses of Ang II (20–320 ng/kg) respectively *in vivo*. The magnitude of CV reactivity observed in HSD, UNX was similar to that of Ctrl. High salt diet drastically increased the *in vivo* cardiac (Fig 2C) and vascular (Fig 2D) reactivity to exogenous Ang II at all the doses in UNX+HSD compared to that of the Ctrl and UNX.

### LTCCs upregulation by high salt in heart and vasculature are involved in the increased Ang II- mediated cardiovascular reactivity

We thought of quantifying calcium signaling, to a certain extent, because calcium ions are crucial to the functioning of heart to contract and relax in a programmed process called excitation-contraction coupling [26]. Diltiazem (DTZ), an L-type calcium channel (LTCC) antagonist which exerts action both on cardiac and smooth muscle was used to get an insight into the expression of LTCCs in both heart and blood vessel. No deviation of ΔLVSP and ΔMAP in response to diltiazem was observed in UNX indicating no change in the expression of LTCCs. HSD rats showed increased ΔLVSP in comparison to Ctrl. Upon DTZ infusion, UNX+HSD rats displayed marked drop in the LVSP and MAP levels compared to Control and UNX indicating the upregulation of LTCCs in heart and vasculature respectively (Fig 2E). Ang II was also co-administered with DTZ, to determine the LTCCs activation coupled with AT1R [27]. Ang II overcame the calcium channel blockade activity of DTZ as evident from the increased ΔLVSP and ΔMAP in UNX+HSD in both cardiac and vascular and HSD in cardiac alone (Fig 2F).

### High salt feeding increases AT1R in the heart and aorta of rats

**Fig 3:**
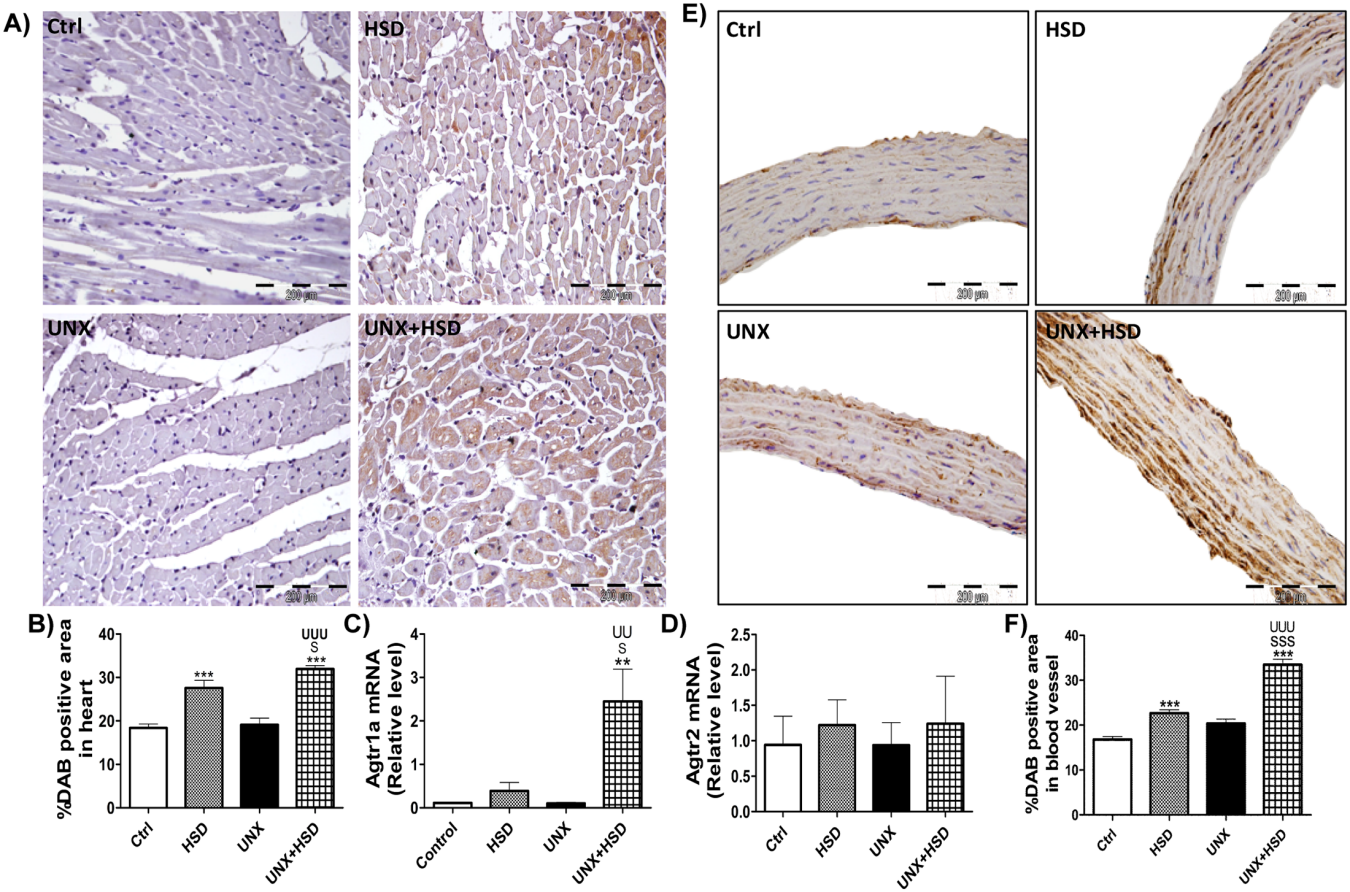
AT1 and AT2 receptor expression at protein and mRNA levels. A, B) Representative photomicrographs (400X) and quantification of AT1R-positive area in heart. C, D) (mRNA) *Agtr1a* and *Agtr2*, which encode AT1 and AT2 receptors respectively in heart. E, F) Representative photomicrographs (400X) and quantification of AT1R-positive area in aorta. N = 3; **P<0.01, ***P<0.001 vs. Control; ^S^P<0.05, ^SS^P<0.01 vs. HSD; ^UU^P<0.01, ^UUU^P<0.001 vs. UNX.

To evaluate whether the enormously increased *in vivo* cardiac and vascular reactivity to Ang II is because of upregulated AT1R, we checked its expression in heart and aortic musculature using immunohistochemistry. No change in the expression of vascular and cardiac AT1R was observed in UNX compared to Ctrl. We observed that AT1 receptor significantly upregulated in the heart and aorta of normal and uninephrectomized rats fed with high salt as evident from the increased DAB-positive area (Fig 3A and 3E). In order to further validate the results of immunohistochemistry, we performed real time PCR of *Agtr1a* and *Agtr2* mRNAs, which encode angiotensin II type 1 (AT1R) and type 2 (AT2R) receptors respectively in the heart. We found that the mRNA expression of AT1R in UNX+HSD heart increased substantially matching with AT1R in immune-stained heart sections (Fig 3C). However, no change was observed in the expression of *Agtr2* mRNA in any of the groups (Fig 3D).

### High salt feeding initiates hypertrophy and fibrosis of kidney, heart and aorta

**Fig 4:**
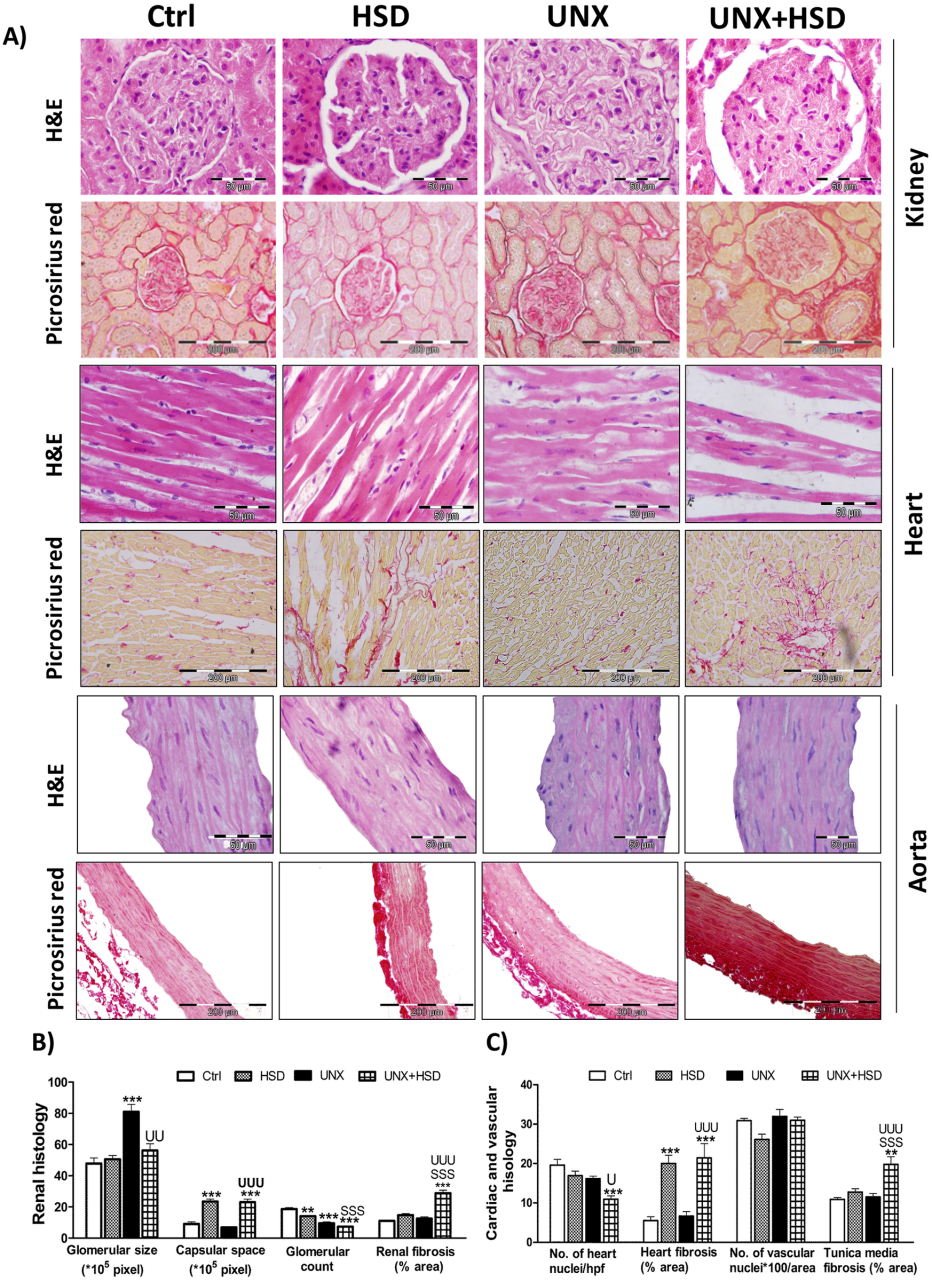
Histopathology of kidney, heart and aorta. A) Representative photomicrographs (1000X, 400X) of H&E- and picrosirius red staining of kidney, heart and aorta. B) Quantification of glomerular size, capsular space, glomerular count, renal fibrosis. C) Quantification of number of heart nuclei per high power focus, cardiac fibrosis, number of nuclei in aorta per unit area and vascular fibrosis in tunica media. N = 3; **P<0.01, ***P<0.001 vs. Control; ^SSS^P<0.001, vs. HSD; ^U^P<0.05, ^UU^P<0.01, ^UUU^P<0.001 vs. UNX.

Uninephrectomy significantly increased the glomerular size and decreased glomerular count compared to that of Ctrl. The capsular space between the Bowman’s capsule and the glomerulus and fibrosis in the kidney of UNX is same as that of Ctrl. UNX+HSD group characterized the features of both high salt diet and uninephrectomy in terms of capsular space and glomerular size respectively and also exhibited highest fibrosis compared to that of Ctrl, HSD and UNX. Nuclei count in heart showed a remarkable decrease in the UNX+HSD group compared to that of Ctrl and UNX. High salt diet augmented fibrosis in HSD and UNX+HSD groups compared to that of Ctrl and UNX groups respectively in the heart (Fig 4). Nuclei count in aorta did not change in any of the groups and high salt diet markedly elevated fibrosis in UNX+HSD compared to that of all the remaining groups (Fig 4).

### Expression of AKT, SERCA and AMPK are altered by high salt feeding

**Fig 5:**
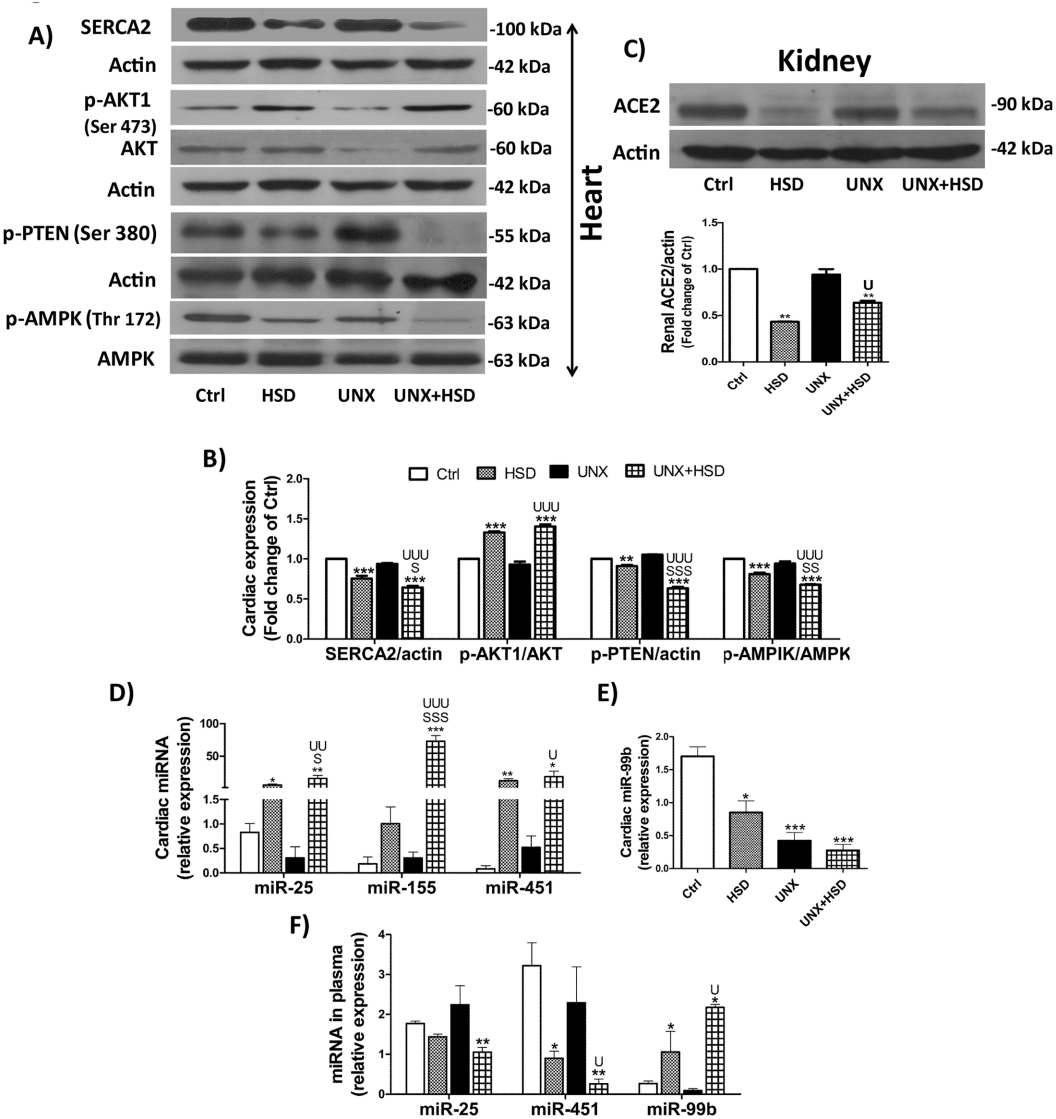
Western blotting and miRNA RT-qPCR. 60 μg of protein was loaded per each lane. To ensure the correct position of the protein of interest, a pre-stained protein marker (Invitrogen Novex^®^ Sharp Pre-stained Protein Standard, Cat # LC5800, Thermo Fisher Scientific) was run along with the samples in the gel. The results were expressed as -fold change over control rats. A) Immunoblots of heart. B) Quantification of western blots of SERCA2 (normalized with actin), p-AKT1 (Ser 473) (normalized with AKT) p-PTEN (Ser 380) (normalized with actin) p-AMPK (normalized with AMPK). C) Immunoblot of renal ACE2 (normalized with actin). D), E) Relative levels of miR-25, miR-155, miR-451 and miR-99b in heart normalized with sn-RNU5G; F) Relative levels of miR-25, miR-451 and miR-99b in plasma normalized with miR-30e. N = 3–5.*P<0.05, **P<0.01, ***P<0.001 vs. Ctrl; ^S^P<0.05, ^SS^P<0.01, ^SSS^P<0.001 vs. HSD; ^U^P<0.05, ^UU^P<0.01, ^UUU^P<0.001 vs. UNX.

No change in the expression of p-AKT, SERCA, p-PTEN and p-AMPK was observed in UNX compared to Ctrl. Reduced expression of SERCA was observed in all the animals fed with high salt diet and UNX+HSD displayed significant decrease compared to UNX and HSD (Fig 5A and 5B).

Increased AKT and decreased AMPK contribute to pathological cardiac hypertrophy in adult hearts [28, 29]. Our results show marked increase in the expression of active form of AKT1 in the animals fed with high salt diet i.e., HSD and UNX+HSD. PTEN is an upstream phosphatase which targets p-AKT and its expression was found to be decreased significantly in the animals (HSD, UNX+HSD) where the surge of p-AKT was observed. Phosphorylated AMPK was significantly reduced in the hearts of normal and uninephrectomized animals fed with high salt (Fig 5A and 5B). ACE2, the protective component of RAS, was significantly dampened in the kidneys of high salt fed rats-HSD and UNX+HSD compared to Ctrl and UNX (Fig 5C).

### High salt feeding alters micro RNA levels (miR-25, -155, -99b, -451) which regulate SERCA, AKT and AMPK

As we observed changes in the protein expression of SERCA, AKT and AMPK, we questioned ourselves whether there would be change in the upstream epigenetic miRs regulating the expression of these proteins and proceeded further to analyse the miRs-25, -99b, -155, -451 in heart. miR-25, which targets SERCA2 [14], was found to be increased in the animals fed with high salt diet. miR-155 and miR-451 upregulated in HSD and UNX+HSD rats (Fig 5D). miR-99, which regulates the expression of AKT [30] along with miR-155 [31], got downregulated in HSD, UNX and UNX+HSD (Fig 5E). miR-25, -155, -451 were unaltered in UNX compared to Ctrl. The reason for downregulation of miR-99b in UNX is not known.

To check whether the pattern of miRs obtained in heart is correlating with that of plasma or not, we assessed the levels of miR-25, -99b, -155, and 451 in plasma. miR-155 was not detected in plasma may be because of its low level of expression. miR-25 and 451 were decreased and miR-99b increased in the plasma of HSD and UNX+HSD groups compared to their controls—Ctrl and UNX respectively (Fig 5F).

## Discussion

Consumption of high sodium in the form of high dietary intake of salt (sodium chloride) has increased enormously across the globe posing the people at risk for both pressure-dependent and independent deleterious effects on various systems. With the increasing demand of renal transplants for patients suffering from ESRD, renal carcinoma etc, the numbers of live kidney donors are also rising alarmingly. To get an insight into the CV and renal functions of such live kidney donors consuming high salt diet, the present study was carried out to investigate the effect of high salt diet on the cardiac, vascular and renal functions in uninephrectomized rats with emphasis on renin angiotensin system (RAS) and epigenetic alterations. High salt elicited cardiovascular and renal-dysfunction and fibrosis, independent of hypertension, in binephric and uninephrectomized rats. To the best of our knowledge, we are the first to investigate the effect of high salt feeding on epigenetic alterations, the levels of tissue and circulating miRNAs and changes in local & systemic RAS in uninephrectomized rats. Our work is different from previous work [32, 33] as we initiated high salt diet feeding after a certain period of normal diet feeding post uninephrectomy, and hence mimics the clinical setting of live kidney donors in a better way.

In compliance with the previous reports in animals [34, 35] and humans [36], we did not observe any anomalies in the kidney function tests as long as the animals were on normal pellet diet. However, inclusion of high salt diet resulted in abnormal kidney function. It is well established that the renal hypertrophy of the remnant kidney after the ablation of the other kidney is compensatory (adaptive/physiological) but not pathological [37] and this phenomenon is independent of calcineurin, transforming growth factor-β1, reactive oxygen species, NAD(P)H oxidase 4 and local RAS components [38], all of which are proved to be upregulated in diabetic nephropathy or in other forms of renal injury. UNX+HSD exhibited highest fibrosis and capsular space compared to that of Ctrl, HSD and UNX. The extent of derangement of biochemical, morphometric parameters and most other molecular intricacies was highest in UNX+HSD indicating that high salt diet worsens end-organ damage in uninephric condition than that of binephric condition, a proof implicating that uninephrectomy sensitizes the animal for high salt dietary intervention.

Contrary to the generalised notion of salt-sensitive hypertension, only one-third of the humans show salt-sensitivity to blood pressure [39]. In line with other preclinical reports [40, 41], we also did not observe any change in SBP by high salt diet. Regulation of blood pressure is pleiotropic. Unchanged SBP in UNX+HSD can be attributed to the significant decrease in plasma Ang II levels (depressed systemic RAS) and a commensurate surge in the *in vivo* cardiovascular reactivity to Ang II (mediated by increased AT1R) and “pressure natriuresis” [42], which was observed in our study in the form of increased sodium in urine. Despite high salt intake, hypertension does not arise until ‘equilibrium between local and systemic arms of RAS’ [40] and ‘renal excretion of excess sodium from the body’ [42] are not hampered. Though hypertension was not observed in our study, we observed pressure-independent deleterious effects on cardiac, vasculature and renal tissue. High salt is known to exert pressure-independent deleterious effects on cardiovascular and renal systems [9]. Baroreflex sensitivity (BRS) is a measure of the development and progression of CVDs [25]. It is the compensatory feedback mechanism instigated in response to change in blood pressure i.e., heart rate (HR) is decreased by the activation of parasympathetic nervous system in response to increased blood pressure; HR is increased by the activation of sympathetic nervous system in response to decreased blood pressure. We provide evidence that the parasympathetic component of BRS is significantly dampened in UNX+HSD group indicating the development of CV dysfunction.

Alterations in cardiovascular reactivity to Ang II can be explained by assuming that Ang II binds to AT1R and mediates vasoconstriction via calcium mobilization by directly transactivating LTCCs, store (sarcoplasmic reticulum)-operated calcium influx or indirectly by activating myosin phosphatase pathways-RhoA/Rho-kinase pathway [43, 44]. Our data shows AT1R is upregulated in heart and aorta of HSD and UNX+HSD rats, suggesting us that activation of local RAS is responsible for increased cardiac and vascular reactivity to Ang II. The same was confirmed from the increased expression of AT1R at protein and mRNA levels in heart. Concurrent with the increased expression of AT1R in heart, aorta and reduced expression of ACE2 in kidney, we observed cardiac, vascular aortic and renal fibrosis in high-salt fed uninephrectomized animals. Activation of any of the components of the pressor arm [AngII (angiotensin II)/ACE (angiotensin-converting enzyme)/AT1Rs (AngII type 1 receptors)] of renin angiotensin system (RAS) instigates cardiovascular and renal fibrosis [45–47]. Further, with a specific L-type calcium channel blocker, diltiazem (DTZ, 160 μg/kg), UNX+HSD animals displayed a significant dip in the LVSP and MAP indicating the upregulation of LTCCs in both cardiac muscle and vascular smooth muscle respectively. LTCCs upregulation was reported in hypertrophic cardiomyopathy, dysrhythmias and failing heart [48]. The extent of LTCCs upregulation in HSD and UNX+HSD was profound that for the given dose of DTZ 160 μg/kg + Ang II 160 ng/kg, some left over LTCCs (even after blockade by diltiazem) are still reactive to Ang II.

We also observed increased left ventricular end diastolic pressure (LVEDP) in normal (HSD) as well as in uninephrectomized rats (UNX+HSD) fed with high salt diet. LVEDP reflects cardiac compliance and is elevated in LV disease associated with or without reduced LV ejection fraction. Increased LVEDP can be explained by decreased expression of SERCA2 [the sarco/endoplasmic reticulum (SR/ER) Ca^2+^ ATPase required for pumping back of cytosolic Ca^2+^ into the lumen of SR/ER to elicit the contraction phase of next cardiac cycle], observed in HSD and UNX+HSD. Declined cardiac function in heart failure is attributed to decreased SERCA2, which in turn is due to increased endogenous miR-25 in mice and humans [14]. Hence, we checked the expression of miR-25, which targets SERCA2 in the heart and interestingly, we observed its level increased in HSD and UNX+HSD demonstrating the involvement of miR-25 in escalating LVEDP in these groups. Several reports suggest reduced activity and expression of SERCA leads to increased LVEDP in diabetic cardiomyopathy and heart failure [49, 50].

Individuals with acute or chronic kidney disease are predisposed to CVD and heart failure via activation of RAS and sympathetic nervous system [51]. We also observed cardiac fibrosis in HSD and UNX+HSD, which supports our data of increased cardiac reactivity to angiotensin II. Cardiac fibrosis was associated with corresponding alterations in the expression of p-AKT and p-AMPK. Increased p-AKT [28] and decreased p-AMPK [52] were positively correlated to cardiac hypertrophy in experimental animal studies. We were interested to check whether the alterations in the expression of these proteins are epigenetically regulated and explored, from the literature, the miRNAs that regulate directly or indirectly the expression of SERCA, AMPK, AKT to be miRs-25, 451, 155 and 99b. It has been reported that miR-155 downregulates the expression of PTEN, which in turn dephosphorylates p-AKT [31]. In our study in cardiac tissue, increased level of miR-155 downregulated PTEN, augmenting p-AKT in HSD and UNX+HSD animals. In the experimental model of wound healing, miR-99b was shown to affect the expression of AKT [30]. Level of miR-99b coincided with the level of p-AKT1 in heart of HSD, UNX+HSD rats. In glioma cells, miR-451 targets CAB39 [53], a binding partner of LKB1 [54], which in turn phosphorylates and activates AMPK [55]. Our data also shows a surge of miR-451 and a proportionate dip in p-AMPK in the heart of HSD and UNX+HSD animals.

To utilize epigenetic alterations as biomarkers for cardiac dysfunction, circulating levels of microRNAs, were also measured in plasma. Several reports indicate both positive [56] as well as negative [57] correlation between the tissue and circulating miRs. However, we failed to observe any positive correlation between the tissue and circulating levels of miR-25, -99b and -451 in our study. Most plausible explanation for this is that some microRNAs are retained/secreted selectively by the affected tissue to contribute to the pathophysiology of disease.

This study identifies that epigenetic alterations by high salt intake culminate in cardiac injury not only in normal (binephric) people but also in live kidney donors and CV & renal injury is worsened more in the latter than the former. Our results also indicate that live kidney donors do not face any major CV and renal dysfunction as long as they are on normal diet but can be predisposed to it upon excursion from normal diet. Contributing factors for cardiac dysfunction include epigenetic alterations and local RAS activation.

## Conclusion

**Fig 6:**
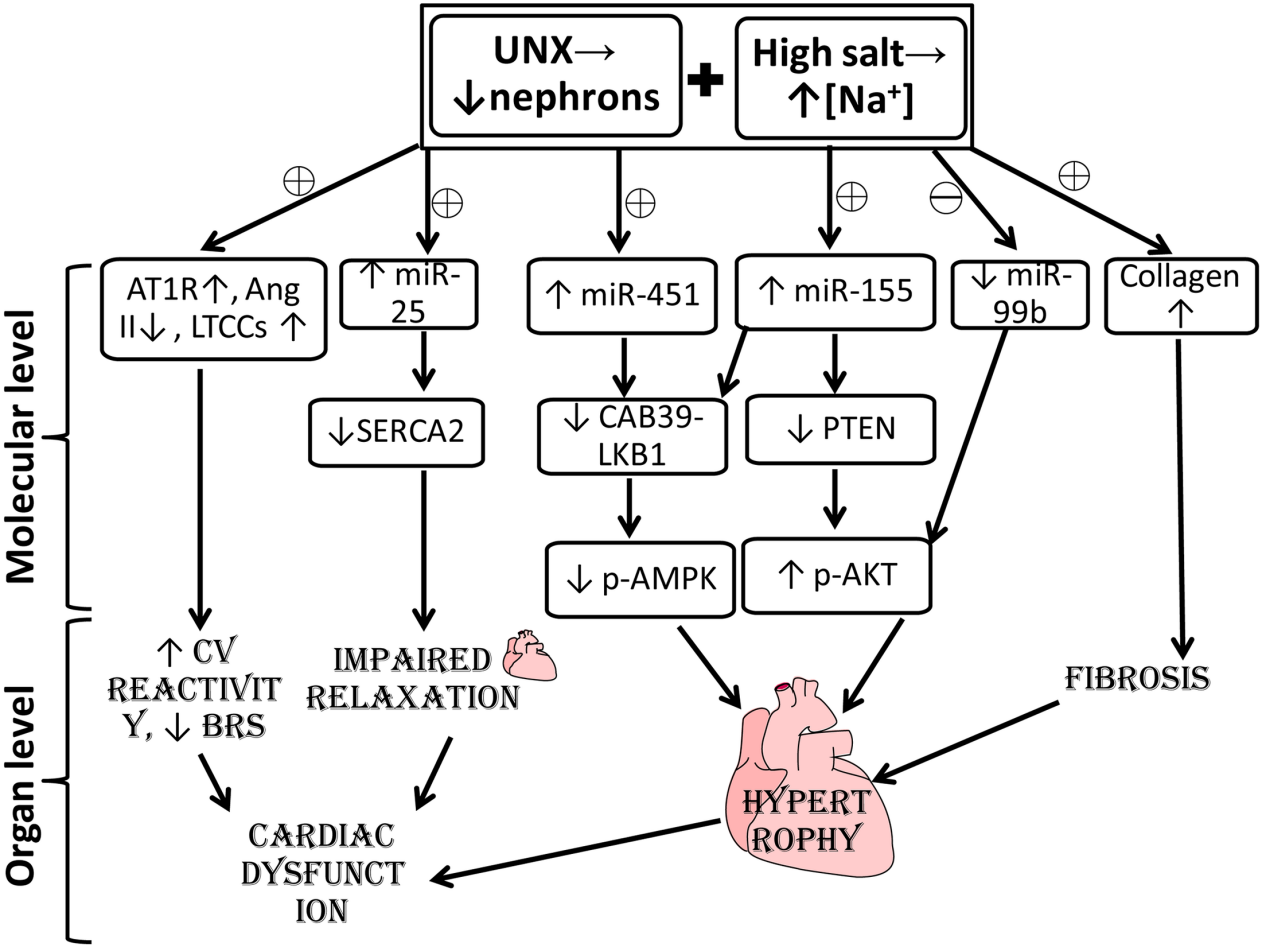
Probable mechanism showing high salt diet intake evokes cardiac dysfunction in uninephrectomy. UNX combined with HSD intake upregulates AT1R, LTCCs leading to increased cardiovascular reactivity and decreased BRS; upregulates miR-25, miR-155 and miR-451 and downregulates miR-99b affecting SERCA2, AKT and AMPK leading to impaired excitation-coupling cycle, fibrosis and hypertrophy culminating in cardiac dysfunction.

Effect of high salt diet on the quality of life of kidney donors is largely unknown. In the present study, we show that high salt diet feeding led to cardiovascular and renal dysfunction in uninephrectomized rats implicating epigenetic alterations (microRNAs (miRs)) and renin angiotensin system (RAS). In a nutshell, our study demonstrated that uninephrectomy *per se* caused no adverse effects, but sensitized the animals to dietary manipulation (high salt diet feeding) culminating in exacerbated cardiac, vascular and renal dysfunction manifested by decreased baroreflex sensitivity, increased *in vivo* cardiovascular reactivity to Ang II and fibrosis in cardiac, vascular and renal tissue. This cardiac dysfunction is attributed to the activation of local RAS, altered cardiac miRNA-25, -99b, -155, -451 and their corresponding targeted proteins—SERCA2, p-AKT, and p-AMPK (Fig 6). Since the pattern of circulating miRs showed a pattern exactly opposite to that of the heart, caution must be exercised in utilizing them as clinical biomarkers.

## Abbreviations

ΔHR: change in heart rate
ΔLVSP: change in left ventricular systolic pressure
ΔMAP: change in mean arterial pressure
Ang II: angiotensin II
AT1R: angiotensin II type 1 receptor
BRS: baroreflex sensitivity
CV: cardiovascular
CVD: cardiovascular diseases
DTZ: diltiazem
LTCC: L-type calcium channels
RAS: renin-angiotensin system
